# Vanillin Alleviates High Fat Diet-Induced Obesity and Improves the Gut Microbiota Composition

**DOI:** 10.3389/fmicb.2018.02733

**Published:** 2018-11-13

**Authors:** Jielong Guo, Xue Han, Jicheng Zhan, Yilin You, Weidong Huang

**Affiliations:** College of Food Science and Nutritional Engineering, Beijing Key Laboratory of Viticulture and Enology, China Agricultural University, Beijing, China

**Keywords:** vanillin, obesity, inflammation, gut microbiota, short chain fatty acids

## Abstract

Vanillin, a simple phenolic compound, exists marginally in some plants and can be produced by microbes. This study uses high-fat-diet (HFD) induced obese mice to study the effect of vanillin on obesity and obtain positive results. First, both body and adipose tissue weight are reduced. Second, the blood properties signaling certain disorders such as ALT, LDH, glucose, cholesterol, LDL-C, TG and HDL-C are ameliorated and both insulin sensitivity, and glucose tolerance are improved. Third, vanillin reduced elevated levels of inflammatory factors including LPS, IL-6, and TNF-α in plasma and liver tissue resulting from obesity. Finally, the production of short chain fatty acids (SCFAs) is enhanced. Additionally, study results demonstrate that vanillin significantly alleviates obesity-related gut microbiota (GM) disorders including the decrease of alpha- and beta-diversity. Furthermore, vanillin reduces the abundance of Firmicutes phylum, increases the richness of Bacteroidetes and Verrucomicrobiota phyla, and inhibits the expansion of the lipopolysaccharide (LPS)-producing bacteria *Bilophila* genus and the H_2_S-producing bacteria *Desulfovibrio* genus.

## Introduction

Obesity rates have surged in recent years, and with them, serious health concerns like type 2 diabetes mellitus (T2DM) and cardiovascular disease (CVD). Researchers have been looking for convenient, safe, and effective methods to prevent and treat obesity and the related metabolic syndromes (Abarca-Gómez et al., 2017). In the early 1960s, Greece and southern Italy captured the research community’s attention with the discovery that populations in this region display the most extended lifespan and lowest risk of metabolic diseases like CVD (Willett et al., 1995; Paletas et al., 2010). They attributed this finding to traditional Mediterranean dietary habits, including a focus on plant-based foods (such as fruits, vegetables, nuts, and cereal), fish and lean meat, as well as moderate wine consumption. Fittingly, this unique diet was dubbed the Mediterranean Diet. Examination of people following this diet in America (Rumawas et al., 2009), France (Kesse-Guyot et al., 2013), and the Canary Islands (Alvarez Leon et al., 2006) found a lower risk of obesity, CVD, and hypertension, compared to those in the same areas who did not adhere to the diet. Long-term epidemiological and preclinical studies suggest that the phenolic components in fruits, vegetables, and wine may be critical to these positive outcomes (Cuevas et al., 2000; Hansen et al., 2005; Pignatelli et al., 2006; Tresserra-Rimbau et al., 2015). Researchers believe that the aforementioned groups’ intake of total polyphenols (PPs) was about 1 g per day, depending on dietary habits (Pinto and Santos, 2017). Most of the dietary PPs cannot be absorbed directly in the small intestine until GM metabolize them into smaller molecular units in the large intestine (Williamson and Manach, 2005). The capacity and products of metabolizing PPs vary substantially between microbes (Dudachodak et al., 2015). Moreover, the diversity of GM varies considerably from individual to individual, (Costello et al., 2009) leading to differences in the ability of individuals to metabolize PPs, thereby affecting the role of PPs in different people (Tomásbarberán et al., 2014; Wu et al., 2016).

The human GM contains approximately 1000 species of microbes, including bacteria, fungi, viruses, protozoa, and archea (Rook et al., 2017). In a healthy state, anaerobic bacteria like Firmicutes and Bacteroidetes phyla inhabit the intestine and can nourish and help maintain the human body. A balanced GM is an asset, providing SCFAs and vitamins, regulating immune system function, and preventing pathogenic microbes from invading the body (Kundu et al., 2017). The GM composition in HFD induced and genetically (*ob/ob*) obese mice are significantly different from that of healthy mice. These results are mainly due to elevated levels of Firmicutes and Proteobacteria phyla, and a decrease in both Bacteroidetes phylum and *Akkermansia* genus (Turnbaugh et al., 2006). The same is true in obese humans. However, weight loss can restore the unbalanced GM (Liu et al., 2017). Numerous animal studies demonstrate that PPs can treat obesity and improve the resultant metabolic syndromes in HFD-induced and *(ob/ob)* obese mice, including hyperglycemia, insulin resistance (IR), and inflammation, as well as restore the GM balance (Axling et al., 2012; Roopchand et al., 2015; Ogura et al., 2016). However, crowd tests showed inconsistent results: many studies employing fruit juices/extracts (which contain abundant PPs) found that they can alleviate hyperglycemia and IR in people suffering from T2DM, (Chiva-Blanch et al., 2012; Saez-Lopez et al., 2017) while other studies found no significant effect (Botden et al., 2012; Woerdeman et al., 2018). Despite the differences in PPs and experimental subjects (such as age, health condition, and genotype) charted in these studies, the variance in how the GM metabolizes PPs may contribute even more to the inconsistent results between subjects. Due to the complexity of natural dietary PPs (Rong, 2010) and the differences in individuals’ GM polyphenol metabolism, (Tomásbarberán et al., 2014; Wu et al., 2016) research into polyphenol function and application is difficult. Instead, studying small metabolites of PPs resulting from the GM metabolic process may be more promising.

The molecular structure of vanillin is less complicated than most of the complex natural PPs. Furthermore, the microbial metabolites (vanillic acid and p-hydroxyphenylpropionic acid) are present in larger quantities (Huang et al., 1993; Narbad and Gasson, 1998). Vanillin exists naturally in various plants and can be produced by microbes from cinnamic acids (Liao et al., 2013). Although vanillin is often used as a flavor compound in the food industry, experiments demonstrate that it can also enhance appetite (Ogawa et al., 2018) and alleviate inflammation (Cheng et al., 2017) and anxiety (Wang et al., 2017). The hypoxia state of the large intestine is crucial in maintaining optimal health in mammals. However, this state is usually disturbed in the presence of disorders such as obesity and inflammation which are represented by the bloom of facultative anaerobic microbes such as phylum Proteobacteria (Byndloss and Baumler, 2018). Vanillin displays moderate reducibility, (Burri et al., 1989) and as such, it is speculated that it may have the capacity to restore balance following intestinal distress. Therefore, this study includes several aims. The first goal is to examine the influence of vanillin on the GM of experimental mice, particularly phylum Proteobacteria. The second objective is to study the effect of vanillin on diet-induced obesity and related syndromes. Lastly, the research intends to investigate the correlation between vanillin, GM and obesity. This study employed C57BL/6J mice fed a HFD to accomplish these goals.

## Experimental

### Chemicals

Vanillin and other chemical reagents were all purchased from Sigma-Aldrich (St. Louis, MO, United States) unless otherwise specified.

### Methods

#### Animals and Diets

Male C57BL/6J mice, purchased at 21 days of age (Vital River Laboratory Animal Technology. Co. Ltd., China), were housed individually in standard conditions (12/12 h light-dark cycle, humidity at 50 ± 15 %, temperature 22 ± 2°C) and were fed with standard laboratory chow for 1 week. After a 7 days adaptation period, the mice were randomly assigned to three groups (*n* = 7–8) including (1) CHOW group fed a standard rodent chow diet (3.85 kcal/g, 10% energy from fat), (2) HFD group fed a HFD diet (4.73 kcal/g, 60% energy from fat), and (3) Va group fed a HFD diet (4.7 kcal/g, 60% energy from fat, containing 0.1% vanillin, m/m). The dosage of vanillin is referred to in Liao’s research who used caffeic acid to treat obesity (Liao et al., 2013).

The food (Huangfukang, China) used in this study was sterilized using radiation (25.0 kGy). Food and water were provided *ad libitum*, and the food was recorded every 3 days. Body weight was recorded weekly. Following a 14-week experimental period the mice fasted for 12 h, and plasma was collected by eyeball extirpation. The contents of the colon, rectum, and caecum were collected, pooled and stored at -80°C for further analysis. The weight of the liver, inguinal white adipose tissue (iWAT) and epididymal white adipose tissue (eWAT) were measured. Tissues were preserved under -80°C for gene expression and the liver was used for Oil Red-O staining.

The guidelines of the Institute regarding the care and use of laboratory animals were followed. This study was approved by the Animal Experiment Committee of the College of Food Science and Nutritional Engineering at China Agricultural University.

#### Glucose and Insulin Tolerance Tests (GTT and ITT)

A GTT was performed on 12-week old mice (after 9 weeks mice of the HFD and Va groups changed to HFD) after a 16 h fast. Glucose concentrations were measured in blood collected by venous bleeding from the tail vein before and 15, 30, 45, 60, 90, and 120 min after an intraperitoneal injection of 1.5 g/kg body weight glucose, using a Roche Diabetes Care glucometer (Roche, Germany). An ITT was conducted on 13-week old mice after a 6 h fast. Glucose concentrations were measured in the blood collected by venous bleeding before and 15, 30, 45, and 60 min after the injection of insulin (Novolin, 30 R, 1.0 U/kg body weight).

#### Magnetic Resonance Imaging (MRI)

An MRI was executed after 11 weeks’ treatment in the Institute of Laboratory Animal Sciences, CAMS & PUMC, China. Mice were anesthetized with 2% isoflurane before and during the experiment. The MRI images were analyzed by utilizing Argus software.

#### Quantitative Real-Time PCR (qPCR) Analysis

The total RNA was extracted using the TRIzol^TM^ Regent (Invitrogen) according to the manufacturer’s instructions. Reverse transcription of the total RNA (2.5 μg) was performed with a high-capacity cDNA reverse transcription kit (Promega Biotech Co., Ltd). The qPCR reactions were run in duplicate for each sample and analyzed in a LightCycler 480 real-time PCR system (Roche). Data were normalized to the internal control actin and analyzed using the ΔΔCT method (Aguilera et al., 2009). The expression of inflammatory factor genes including the tumor necrosis factor (TNF-α) and interleukin 6 (IL-6) in the liver and adipocyte tissue, as well as bacterial load were determined through qPCR (Primers used are shown in Table 1).

**Table 1:** Quantitative PCR primers used in this study.

Gene	Forward	Reverse
IL-6	5′-AGACAAAG CCAGAGTCCTTCAG	5′ -GCCACTCC TTCTGTGACTCCAG
TNF-α	5′-CCAACAAG GAGAAGT	5′-GTATGAAGT GGCAAATCG
16s universal	5′-ACTGGGTA TAAAGNG	5′-TACCAGGG TCTCTAATCC
16s rRNA (V3-V4)	5′-AATGATAC GGCGACCACCGAG ATCTACACTATGG TAATTGTGTGCCA GCMGCCGCGGTAA	5′ -CAAGCAGA AGACGGCATAC GAGATXXXXXX XXXXXXAGTCAGTC AGCCGGACTACHVG GGTWTCTAAT

For the quantification of the bacterial load, the total bacterial DNA was isolated from the weighed pooled samples employing a QIAamp DNA Stool Mini Kit (Qiagen). The DNA was then subjected to qPCR using a QuantiFast SYBR Green PCR kit (Biorad) with universal 16SrRNA primers (Table 1) (Cho et al., 2012). Results were expressed as bacteria number per g of samples, using a standard curve which was built using *Bifidobacterium longum*.

#### Oil Red-O Staining

Oil Red-O staining was performed as described by Ross et al. (1999). Briefly, liver slices were washed with phosphate-buffered saline, fixed in 3.7% formaldehyde for 2 min, washed with H2O, incubated with Oil Red-O solution for 1 h at room temperature, and then washed with H2O.

#### Plasma Parameters

The plasma biochemical parameters, including alanine transaminase (ALT), glucose, cholesterol, triglyceride (TG), high-density lipoprotein cholesterol (HDL-C), low-density lipoprotein cholesterol (LDL-C), and lactate dehydrogenase (LDH) were determined by a 3100 Clinical Analyzer (Hitachi High-Technologies Corporation, Japan). Plasma inflammation factors, including IL-6, TNF-α, and LPS, were determined by enzyme-linked immunosorbent assay (ELISA) kits (ThermoFisher, United States), according to the operating instructions.

#### Determination of the Concentration of SCFAs

The SCFAs, including acetate, propionate, *n*-butyrate, iso-butyrate, and *n*-valeric acid were determined by GC-MS as previously described (Han et al., 2018). Briefly, 65 mg fresh caecum contents were placed into a 2 mL tube, freeze-dried for 3.5 h. This process was followed by the addition of water, 50% H_2_SO_4_, and ether, with volumes of 0.8 ml, 0.2 ml, and 1.0 ml, respectively. The mixture was extracted and agitated for 15 min at 4°C, and centrifuged for 10 min at 12000 *g*, 4°C. CaCl_2_ was added to the supernatant to absorb water using a 0.22 μm filter before injecting the supernatant into the GC-MS system. Analysis was performed using an Agilent 7890B gas chromatography system, coupled with an Agilent 5977B mass spectrometric detector (HES, GC/MSD, Agilent Technologies, United States). The extract was separated using a J&W Scientific HP-FFAP column (30 m × 0.25 mm × 0.25 μm, from Agilent Technologies). A 1 μl sample was manually injected into a split/splitless inlet (in split mode, 20:1), which was kept at 175°C. Helium was used as a carrier gas at a flow rate of 1 ml/min. The oven temperature was initially set at 90°C, then increased to 150°C at a rate of 12°C/min, then increased further to 220°C at a rate of 20°C/min, and maintained at 220°C for 4.5 min. The temperatures of the transfer line and electron impact (EI) ion source were set at 220 and 230°C, respectively. The solvent delay was set at 3 min. The standard curves of the target SCFAs were designed to quantify the SCFAs.

#### GM Analysis

Total genome DNA was extracted from the pooled samples using the CTAB/SDS method. DNA concentration and purity was monitored on 1% a garose gels and diluted to 1 ng/μl using sterile water. 16S rRNA genes were amplified using the specific primer (Table 1) with a barcode. All PCR reactions were conducted in 30 μL solutions with 15 μL of Phusion^®^ High-Fidelity PCR Master Mix (New England Biolabs, United States), along with 0.2 μM of forward and reverse primers, and about 10 ng of template DNA. Thermal cycling consisted of initial denaturation at 98°C for 1 min, followed by 30 cycles of denaturation at 98°C for 10 s, annealing at 50°C for 30 s, and elongation at 72°C for 30 s and finally, 72°C for 5 min. The PCR products were mixed in equal parts. The mixture was purified using the GeneJET Gel Extraction Kit (Thermo Scientific, United States). Sequencing libraries were generated using the TruSeq^®^ DNA PCR-Free Sample Preparation Kit, by following the manufacturer’s recommendations. Index codes were added. The library quality was assessed on the Qubit@ 2.0 Fluorometer (Thermo Scientific) and the Agilent Bioanalyzer 2100 system. Finally, the library was sequenced on an Illumina HiSeq 2500, and 250 bp paired-end reads were generated.

Paired-end reads from the original DNA fragments were merged by using FLASH, (Magoč and Salzberg, 2011) a high-speed and accurate analysis tool designed to merge paired-end reads when overlaps exist between read 1 and read 2. Paired-end reads were assigned to each sample according to the unique barcodes. Sequences were analyzed using the QIIME (Caporaso et al., 2010) (Quantitative Insights Into Microbial Ecology) software package. In-house Perl scripts were used to analyze alpha- (within samples) and beta- (among samples) diversity. First, reads were filtered by QIIME quality filters. Then, pick_de_novo_otus.py was used to pick operational taxonomic units (OTUs) by creating an OTU table. Sequences with ≥97% similarity were assigned to the same OTUs. Researchers picked a representative sequence for each OTU and used the RDP classifier (Wang et al., 2007) to annotate taxonomic information for each representative sequence.

#### Statistics

All data reported in this paper are expressed as means ± SD. The data was evaluated by a one-way ANOVA followed by Duncan’s significant difference test. All statistics were analyzed by SPSS software and performed with GraphPad Prism 7.

## Results

### Vanillin Alleviates the Metabolic Syndromes Caused by HFD

#### Vanillin Alleviates Obesity Induced by HFD

**FIGURE 1:**
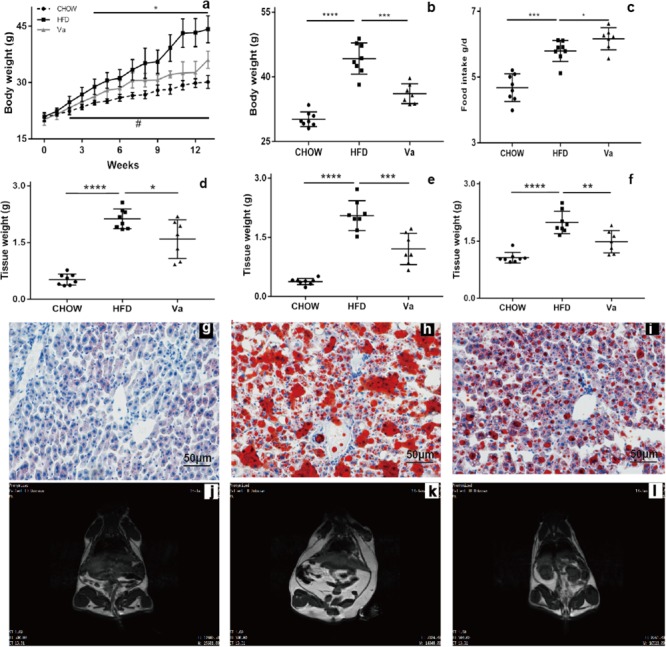
Vanillin ameliorates the obesity and fat accumulation caused by HFD. **(a)** The change in body weight of the mice during the experiment. **(b)** The change in body weight of the mice at the end of the experiment (14 weeks). **(c)** The food intake of the mice. **(d–f)** The tissue weight of eWAT, iWAT, and the liver, respectively. **(g–i)** The Oil Red-O staining of liver samples taken from the CHOW, HFD, and Va groups, respectively (from left to right). Each represents 7–8 samples. **(j–l)** The MRI graphs of the CHOW, HFD and Va groups, respectively (from left to right). The white parts represent the lipids, and each represents 7–8 samples. ^∗^*P* < 0.05, CHOW compared to HFD group, *^∗∗^P* < 0.01, *^∗∗∗^P* < 0.001 and *^∗∗∗∗^P* < 0.0001; ^#^*P* < 0.05, Va compared to HFD group for **(a)**.

HFD-induced obese mice are widely used animal models to study obesity and the related metabolic syndromes. In this experiment, 4 weeks after changing to an HFD, the body weight (BW) of the HFD group was significantly higher than that of both the CHOW and Va groups for the remainder of the experiment (Figure 1a). Results showed that the food consumption of the Va group was slightly higher than that of the HFD group (Figure 1c). At the end of the experiment, the average BW of the HFD group reached 44.32 g, which was 52.15 and 22.90% higher than that of the CHOW and Va groups, respectively (Figure 1b). In addition to BW, the weight of the eWAT, the iWAT, and the livers of the HFD group were significantly higher than those of the CHOW and Va groups (Figures 1d–f). In comparison with the HFD group (*P* < 0.01 for all), significant weight reductions of 21.21, 34.55, and 24.21%, respectively were evident in the eWAT, iWAT and livers of the Va group. Figures 1g–l indicates that vanillin reduces the accumulation of lipids in the entire body, particularly the liver, which is consistent with the weight measured.

#### Vanillin Improves the Blood Biochemical Parameters Related to Metabolic Syndromes

**FIGURE 2:**
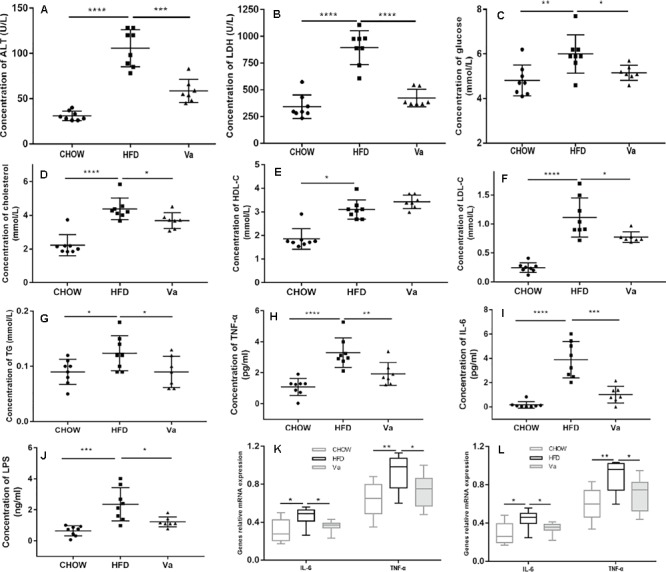
Vanillin improves the blood biochemical and inflammatory parameters related to obesity. **(A–G)** The blood biochemical parameters of the mice. **(H–J)** The inflammatory parameters in the blood of the mice. **(K,L)** The inflammation-related genes relative to mRNA expression of the colon **(K)** and the liver **(L)** *^∗^P* < 0.05, *^∗∗^P* < 0.01, *^∗∗∗^P* < 0.001 and *^∗∗∗∗^P* < 0.0001.

Plasma parameters were tested to investigate the influence of vanillin, the results of which are shown in Figure 2. Mice fed on an HFD (Va and HFD groups) displayed a significant increase of ALT, LDH, glucose, cholesterol, LDL-C, TG, and HDL-C when compared to mice fed the CHOW diet (CHOW group). Conversely, vanillin significantly decreased these parameters, except for the HDL-C levels, which exhibited minimal variance in both the HFD and Va groups.

#### Vanillin Improves IR and Hyperglycemia

**FIGURE 3:**
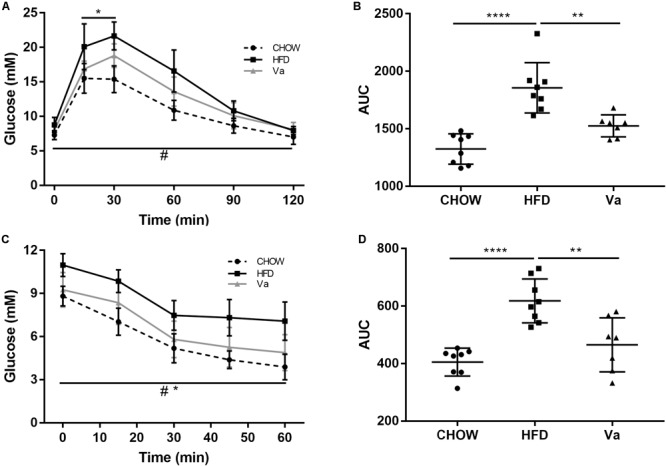
Vanillin alleviates the glucose intolerance and IR of the mice related to obesity. **(A,C)** The changes in blood glucose concentrations during GTT **(A)** and ITT **(C)**. *^∗^P* < 0.05, CHOW compared to HFD group; ^#^*P* < 0.05, Va compared to HFD group. **(B,D)** The area under the curves (AUC) of **(A,C)**, respectively. *^∗∗^P* < 0.01 and *^∗∗∗∗^P* < 0.0001.

ITT and GTT reflect the degree of insulin functionality, as well as glucose tolerance. Mice fed the HFD (HFD and Va groups) show an impaired glucose tolerance, compared to mice fed the CHOW diet (Figures 3A,B). However, the Va group shows a significantly improved glucose tolerance compared to the HFD group (*P* < 0.01). Similar to those of the GTT, the ITT results demonstrated consistency (Figures 3C,D). Mice fed an HFD showed impaired insulin sensitivity, compared to mice fed the CHOW diet. Vanillin significantly improved this concern.

#### Vanillin Improves the Inflammation Triggered by HFD

It is widely accepted that obesity can cause systemic low-level inflammation and increase the concentration of inflammatory factors such as TNF-α and LPS in plasma (Gregor and Hotamisligil, 2010). Figures 2G–I show that compared to the mice in the CHOW group, the plasma concentration of LPS, TNF-α, and IL-6 in the HFD group was significantly increased. Simultaneously, these parameters were substantially elevated in Va group mice, compared to those in the HFD group. Consistent with these results, the expressions of TNF-α and IL-6 in the colon and liver were considerably higher in the HFD group than in the Va and CHOW groups (Figures 2J,K).

#### Vanillin Enhances the Production of SCFAs

**FIGURE 4:**
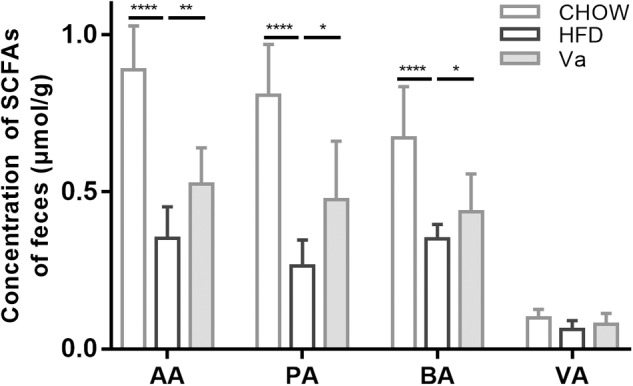
The concentration of SCFAs in the caecal contents. AA, acetate acid; PA, propionate acid; BA, butyrate acid and VA, valeric acid. *^∗^P* < 0.05, *^∗∗^P* < 0.01, *^∗∗∗^P* < 0.001, and *^∗∗∗∗^P* < 0.0001.

Intestinal SCFAs are produced by the anaerobic fermentation of polysaccharides and are crucial to intestinal health. Compared to the HFD group, Figure 4 shows that vanillin substantially increased concentrations of acetate, propionate, and butyrate, previously reduced by the HFD. Conversely, n-valeric acid was not significantly influenced by diet.

### Vanillin Restores the Disturbed GM Caused by HFD

**FIGURE 5:**
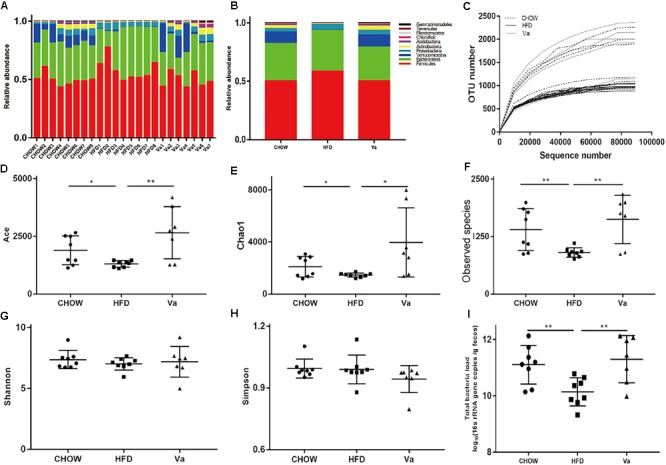
Vanillin (partly) restores the diversity and richness of the GM that is disturbed by obesity. **(A,B)** The relative abundance of the bacteria (top 10 phyla). **(C)** The rarefaction curve during the sequencing. **(D–H)** The α-diversity and the observed species of the GM. **(I)** The bacterial load of the pooled samples was determined by qPCR using universal primers for bacterial 16S rRNA. *^∗^P* < 0.05, *^∗∗^P* < 0.01.

Bacteria composition was analyzed by sequencing their 16s rRNA. Figure 5C shows that the number of species found initially expands but slows down as the depth of the sequencing increases. It remains mostly unchanged when the depth reaches a certain level. These results suggest that the depth of the sequencing is sufficient for subsequent analysis. The abundance of Firmicutes phylum in HFD mice GM composition is significantly higher (*P* = 0.02 for both CHOW and Va groups) than that of the CHOW and Va groups (Figures 5A,B). Facultative anaerobic bacteria such as Proteobacteria phylum is higher in the HFD group compared to that of the CHOW (*P* = 0.07) and Va (*P* = 0.29) groups. However, the Verrucomicrobia phylum of the HFD group is under-represented compared to the CHOW (*P* < 0.01) and Va (*P* = 0.03) groups. Moreover, the richness of Actinobacteria phylum, including the widely used commensal bacteria *Bifidobacteria* which can reduce the production of LPS, is significantly higher in the GM of mice belonging to the CHOW and Va groups. The Chao1 index, representing the richness of the GM, of the HFD group GM is smaller than that of the CHOW (*P* = 0.04) and Va (*P* = 0.02) groups, which shows that vanillin reverses the decreased GM richness caused by the HFD, which is consistent with results of the bacterial loads determined by qPCR (Figures 5E,I). Other parameters reflecting the richness of the GM, including the ace index and observed species, show similar results (Figures 5D,F). In addition to richness, the homogeneity of the GM is not significantly different between the groups, as shown in indexes by Shannon and Simpson (Figures 5G,H).

**FIGURE 6:**
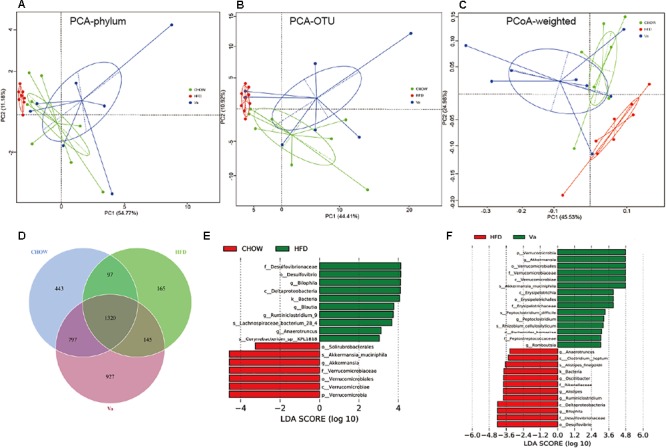
Vanillin partially restores the GM to its normal state. **(A,B)** Principal component analysis (PCA) of the GM at phylum **(A)** and OUT **(B)** levels, respectively. **(C)** Principal coordinate analysis (PCoA) of the GM based on weighted UniFrac distance. **(D)** Common OTU inter groups. **(E,F)** Biomarker (LEfSe) analysis between HFD and CHOW **(E)** and Va **(F)**, respectively. The threshold value of LDA is three.

Figure 6C shows a substantial difference between the composition of GM in the HFD and the CHOW or Va groups. The GM composition of the CHOW and Va groups display similarities based on the weighted UniFrac distance. This finding suggests that the CHOW and Va groups have a similar GM composition. The Venn diagram (Figure 6D) shows similar results: the CHOW and Va groups have about 50% more species in common than the CHOW and HFD groups; species found only in the CHOW and Va groups outnumber those of the CHOW and HFD groups by a factor of eight. Furthermore, similar to results illustrated by Figure 5E, the Venn diagram shows that the GM of the HFD group contained fewer species. As Figures 6A,B indicate, the difference in intra-group subjects in the HFD group is lower than that of the CHOW and Va groups, both in phylum and OTU levels. However, the CHOW and Va groups share a closer connection. Figures 6E,F show that the richness of GM variance between the HFD and the CHOW or Va groups are most prominent in the *Ruminiclostridium* genus (belonging to the Firmicutes phylum), the Deltaproteobacteria class (belonging to the Proteobacteria phylum), and the Akkermansia genus (belonging to Verrucomicrobia phylum). The abundance of the Ruminiclostridium genus and the Deltaproteobacteria class are more pronounced, while the Akkermansia genus is less prevalent in the HFD group compared to the CHOW and Va groups. Moreover, in comparison with the HFD group, vanillin significantly decreases the abundance of the Bilophila genus (belonging to the Proteobacteria phylum), as well as *Clostridium leptum* (belonging to the Firmicutes phylum).

## Discussion

The results show that vanillin significantly reduces HFD-induced obesity, even though the food intake of the Va group was considerably higher compared to the HFD group. The appetizing effect of vanillin was described before (Cox and Blaser, 2013) and was verified by the observations in this study. The abundance of phylum Verrucomicrobia in the GM of the mice was entirely restored by vanillin compared to that of the HFD group. The bloom of phylum Verrucomicrobia is usually accompanied by the improvement of host metabolism. For example, combined with flourishing phylum Verrucomicrobia in the GM, the Roux-en-Y Gastric Bypass (RYGB) can dramatically improve the glucose homeostasis and obesity of individuals suffering from T2DM (Cox and Blaser, 2013). Therefore, as with RYGB, some types of broad-spectrum antibiotics show similar effects (Cox and Blaser, 2013). *Akkermansia muciniphila*, the only representative bacteria of the phylum Verrucomicrobia, accounts for 3∼5% of the GM, and its presence is positively correlated with the consistency of mucus (Zhou, 2017). Studies show that the lack of *A. muciniphila* is a primary characteristic of obesity in humans, as well as in HFD-induced and *ob/ob* obese mice, (Rajilic-Stojanovic et al., 2013; Langille et al., 2014; Zhou et al., 2016) which is consistent with the findings of this research. Supplementing *A. muciniphila* in mice that were fed an HFD can restore its abundance, successfully decreasing the adipose tissue mass and reversing the obesity caused by an HFD (Shin et al., 2014). In addition to directly supplementing one’s diet with living microbes, prebiotics such as fructooligosaccharide (FOS), or certain drugs including metformin and vancomycin, can support the profusion of *A. muciniphila*, and improve obesity, hyperglycemia, and IR caused by an HFD (Zhou, 2017). Consequently, this outcome is similar to the results of this study. Therefore the restoration of phylum Verrucomicrobia or more specifically, *A. muciniphila* by vanillin may be partly responsible for its anti-obesity effect. In addition to vanillin, other PPs present in black tea and wine can further enhance the *in vitro* reproduction of *A. muciniphila* (Kemperman et al., 2013). However, *in vivo* experiment results were inconsistent (Axling et al., 2012; Anhe et al., 2015; Li et al., 2015; Roopchand et al., 2015). Supplementing PPs with Concord grapes and cranberries significantly increased the abundance of *A. muciniphila*, (Anhe et al., 2015; Roopchand et al., 2015) while green tea and pomegranate did not (Axling et al., 2012; Li et al., 2015). The difference is possibly the result of the complexity of natural PPs (Rong, 2010), but could also be due to the varying degrees that the test subjects are able to metabolize PPs (Tomásbarberán et al., 2014; Wu et al., 2016). Nonetheless, these issues limit researchers’ ability to apply PPs to the treatment of obesity and other metabolic syndromes by way of improving the presence of *A. muciniphila.* The same issues do not arise with vanillin, but the benefits remain.

Vanillin ameliorates obesity-related metabolic syndromes, including hyperglycemia, IR, and inflammation. These results are similar to those from studies of other natural phenolic components, such as ferulic acid and caffeic acid in a comparable dosage (Liao et al., 2013; Ilavenil et al., 2017). It is notable that inflammation, IR and obesity are intimately connected (Gregor and Hotamisligil, 2011). However, only recently has GM been recognized as an essential component in this association. Studies show that an HFD can increase both the concentration of LPS and the abundance of LPS-producing bacteria in GM, (Cani et al., 2007, 2008a) which is congruent with the findings of this research (Figures 2J, 6). Furthermore, similar to the induction of an HFD, a subcutaneous LPS injection caused obesity and IR in mice and increased the weight of the adipose tissue and liver (Cani et al., 2007). These results indicate that LPS is a critical factor connecting an HFD and obesity. It appears that reducing the concentration of LPS may be an effective method to control HFD-induced obesity. However, inconsistent and even opposing results exist when studying the association between GM, LPS, and obesity based on the present observations. First, although controversial, the abundance of the LPS-producing gram-negative bacteria phylum Bacteroidetes is generally reduced in the GM of *ob/ob* and diet-induced obesity in mice and obese humans, (Cox and Blaser, 2013; Saad et al., 2016) while the concentration of plasma LPS is higher. Second, treating obesity with prebiotics such as inulin can increase the abundance of phylum Bacteroidetes in the GM, while reducing the concentration of plasma LPS (Zhang et al., 2018). Some theories may explain these paradoxes such as the changes of intestinal permeability influenced by obesity or prebiotics, the LPS-producing abilities of the gram-negative bacteria, or the different immune responses of the various LPS (Cani et al., 2008a, 2009). Some specific LPS-producing gram-negative taxa belonging to phylum Bacteroidetes are indeed closely related to obesity and inflammation such as *Bilophila* genus (Cani et al., 2008b). However, others such as *Bacteroides* genus display a negative correlation to obesity and inflammation (Vatanen et al., 2016). *Bilophila*, a common LPS-producing bacteria, can aggravate the HFD-induced metabolic dysfunction and thrive in the inflamed intestine (Natividad et al., 2018). *Bilophila* is the only taxa belonging to phylum Bacteriodetes that was significantly decreased by vanillin. Therefore, this result may be responsible for the reduction of the plasma LPS concentration, and the improvement of obesity and the related metabolic syndromes.

The hypoxia state combined with thriving colonies of beneficial bacteria in the intestine are crucial to the maintenance of optimal health in mammals (Byndloss and Baumler, 2018). The expansion of facultative anaerobes such as Firmicutes and Proteobacteria phyla prompted by elevated oxygen and oxide levels in the intestine, is a typical feature of intestinal dysbiosis (for example, inflammation) (Rigottier-Gois, 2013). Study shows that HFD can cause the expansion of phylum Proteobacteria (Byndloss and Baumler, 2018), similar to our findings (Figure 5). Compared to the HFD group, vanillin substantially reduced the abundance of the *Desulfovibrio* genus belonging to the phylum Proteobacteria (Figure 5). *Desulfovibrio* is responsible for the production of hydrogen sulfide (H_2_S), and an estimated 60% of the total H_2_S in the colon is attributed to this bacteria (Linden, 2014). H2S can inhibit the mitochondrial respiration of colonic epithelial cells, (Beaumont et al., 2016) while the respiration expends massive amounts of oxygen through the beta-oxidation of butyrate. Therefore, this process reduces the diffusion of oxygen from epithelial cells (ECs) to lumen and contributes to anaerobiosis of the colon (Byndloss et al., 2017). Furthermore, the anaerobic fermentation of polysaccharides is enhanced by anaerobiosis leading to the production of SCFAs, which can inhibit inflammation (Byndloss et al., 2017). Moreover, the reduction of H_2_S-producing bacteria by vanillin enhances the output of SCFAs, thus improving intestinal health and inflammation. Vanillin also significantly decreases the *Rikenella* genus and *Clostridium leptum*, which occur at high levels in inflammatory bowel disease (IBD) patients and HFD-induced obese mice, respectively. The reduction of *C. leptum* is thought to provide protection against obesity and T2DM (Adams and Morrison, 2016; Fransen et al., 2017).

Despite the connection between improved GM and resistant obesity treated with vanillin, the mechanism driving this association remains unclear. For example, is the improvement of GM a cause or a result of resistant obesity subjected to vanillin? Furthermore, should it be a result, what is the mechanism? Therefore, more research is necessary to provide answers to these questions.

## Author Contributions

All authors listed have made a substantial, direct and intellectual contribution to the work, and approved it for publication.

## Conflict of Interest Statement

The authors declare that the research was conducted in the absence of any commercial or financial relationships that could be construed as a potential conflict of interest.
